# Care-related Quality of Life of informal caregivers of the elderly after a hip fracture

**DOI:** 10.1186/s41687-018-0048-3

**Published:** 2018-05-03

**Authors:** Cornelis L. P. van de Ree, Kari Ploegsma, Tim A. Kanters, Jan A. Roukema, Mariska A. C. De Jongh, Taco Gosens

**Affiliations:** 1 Department Trauma TopCare, Elisabeth-TweeSteden Hospital, Tilburg, The Netherlands; 2 Institute for Medical Technology Assessment (iMTA), Erasmus University Rotterdam, Rotterdam, The Netherlands; 3 Center of Research on Psychological and Somatic disorders, Tilburg University, Tilburg, The Netherlands; 4 Brabant Trauma Registry, Network Emergency Care Brabant, Tilburg, The Netherlands; 5 Department of Orthopaedic Surgery, Elisabeth-TweeSteden Hospital, Tilburg, The Netherlands

**Keywords:** Hip fracture, Elderly, Informal care, CarerQoL instrument

## Abstract

**Background:**

Reforms in the Dutch healthcare system in combination with the aging of the population will lead to a strong increase in the demand for informal care in the Netherlands. A hip fracture is one of the most important causes of hospital admissions among frail elderly and informal caregivers experience stress that may have significantly negative impact on the caregivers’ Quality of Life. The purpose of the study was to determine the nature, intensity and the care-related Quality of Life (CarerQoL) of informal caregivers of elderly patients in the first six months after a hip fracture. In this cross-sectional study, were interviewed the primary informal caregivers of patients with a hip fracture about the informal care provided after one, three or six months following the injury. The CarerQoL of the informal caregivers was measured with the CarerQoL-7D instrument.

**Results:**

In total, 123 primary informal caregivers were included. The CarerQoL-7D score was on average 83.7 (SD 15.0) after one, three and six months, and there were no major differences between the measurement time points. The average amount of informal care provided per patient per week was 39.5 during the first six months.

Partners of patients with a hip fracture provided significantly more hours of informal care (*β* 34.0; 95% CI: 20.9 – 47.1). Female informal caregivers stated a significantly lower level of CarerQoL (*β* -7.8; 95% CI: -13.3 – -2.3). Female caregivers were 3.0 times more likely to experience relational problems (aOR 3.02; 95% CI 1.08-8.43). Caregivers provided care at 6 months were associated with physical health problems (aOR 2.54; 95% CI 1.05-6.14).

**Conclusions:**

Informal caregivers, especially partners, are faced with providing care of greater intensity to elderly patients during the first six months after a hip fracture. The CarerQoL was not associated with the intensity of the provided informal care. However, this study shows that a considerable group of informal caregivers for elderly patients with a hip fracture experienced relational, physical and mental health problems that stemmed from providing intensive informal care during the first six months.

**Electronic supplementary material:**

The online version of this article (10.1186/s41687-018-0048-3) contains supplementary material, which is available to authorized users.

## Background

Due to recent reforms in the Dutch healthcare system, the number of elderly people remaining at home longer continues to rise. By 2020, 800 of the 2000 nursing homes in the Netherlands will be closed due to increasingly stringent cost-containment policies involving the Long-Term Care Act introduced in 2015 [1]. The Social Support Act 2015 transferred publicly provided care to the private sector, calling for more self-reliance on the part of citizens and creating a larger role for municipalities in its organization. This led to a reduction in the household support and home care that is provided to patients needing temporary services following hospital discharge, patients with chronic conditions requiring medical services, people with mental or psychological disabilities, and individuals in need of end-of-life care [2, 3]. The main goal of these health-care reforms is to keep care affordable and to increase both the system’s efficiency and its responsiveness to patient needs. These reforms, in combination with the aging of the population, will lead to a strong increase in the demand for informal care in the Netherlands [4, 5].

The current situation shows that informal caregivers are overburdened, and there is increasing awareness that the impact of providing informal care to patients is continuing to grow [6–9]. Earlier research has revealed that informal care affects the well-being of informal caregivers and can lead to personal and social costs. The mortality of older informal caregivers may even increase when they take on the care of their partners [10, 11].

In 2014, in the Netherlands, 20,254 patients were admitted to hospital with a hip fracture, 17,184 of whom were 65 years and older [12]. A hip fracture is one of the most important causes of hospital admissions among the elderly and leads to a loss of independence and Quality of Life (QoL), as well as being associated with a high mortality rate [13, 14]. Therefore, these patients belong to one of the larger groups in society that suddenly need informal care for a shorter or longer period. The recovery process after treatment depends on several aspects, such as comorbidity, the level of activities of daily living (ADL), living environment, cognitive ability and the psychosocial status of the patient [15]. This process can be slow and difficult for dependent elderly patients, and the role of informal caregivers is very important [16]. Informal caregivers not only provide practical help but also offer emotional and psychological support and have a key role in enhancing patient motivation. However, in-depth interviews with 10 informal caregivers providing care to patients with hip-fractures showed that the new caregiver role can be overwhelming. Informal care required management of a multitude of caregiving activities, including assistance in physical care, financial transactions, and placement after discharge from the acute hospital. Furthermore, most caregivers must address quickly changing care needs as the care recipients transition from emergency room to operating room, then to a regular hospital unit, followed by a rehabilitation setting, and then home. Most caregivers take up their role without prior knowledge or experience, and the associated stress may have a significantly negative impact on the caregivers’ QoL [17].

The main purpose of this study was to determine the nature and intensity of informal caregiving and determine the care-related Quality of Life (CarerQoL) of those providing informal care to elderly patients in the first six months after a hip fracture. The second purpose was to examine whether certain informal caregiver or patient characteristics influenced the time investment or CarerQoL of the informal caregiver.

## Methods

### Participants and design

Hip fracture cohort data were derived from the Brabant Injury Outcome Surveillance (BIOS), a prospective cohort study measuring health status (HS) and level of frailty of patients with a hip fracture [18]. One contact person per hip-fracture patient, who was included in the BIOS, was approached by telephone. We used a simple random sampling method where we randomly selected a subset of individuals from the BIOS. Contact persons were approached between January and September 2016 at one, three or six months following a hip fracture in their loved ones. It was a cross-sectional study, and all caregivers participated at one time point only. Contact persons, a family member or an unpaid helper, were asked if they provided assistance with personal care, household chores, nursing, mobility outdoors, logistic- or social activities. The Medical Ethics Committee Brabant approved the study (NW2016-26). Informed consent was obtained from all participants. Caregivers were included if they provided informal care to a hip-fracture patient aged 65 years and older at one, three or six months. Exclusion criteria were (i) patients who did not receive informal care, (ii) patients for whom no informal caregiver was available and (iii) patients who died before the point of measurement.

### Instruments

We obtained patient characteristics from the medical files and the BIOS study. We examined informal caregivers’ socio-demographic and health characteristics through a telephone interview. Informal caregivers’ socio-demographic and health characteristics included age, sex, relationship to the patient, educational attainment, and nature and intensity of the informal care they had provided.

#### CarerQoL

The care-related Quality of Life instrument (CarerQoL-7D) was conducted by a telephone interview and measured CarerQoL in terms of subjective burden and general well-being (Additional file 1) [19]. This questionnaire consists of the CarerQoL-7D and the CarerQoL-VAS (visual analogue scale). The CarerQoL-7D consists of seven items, each covering one dimension of the subjectively experienced impact of informal care (satisfaction, support, problems with daily activities, and financial, relational, mental health and physical health problems). Informal caregivers can indicate for each dimension whether they had experienced ‘no’ problems, ‘some’ problems or ‘a lot’ of problems. The scores were transformed to a scale of 0 (worst informal care situation) to 100 (best informal care situation) using the Dutch CarerQol tariff, in which a higher score represents a better CarerQoL [20]. The CarerQoL-VAS, from 0 (completely unhappy) to 10 (completely happy), measured general well-being in terms of happiness. A second VAS (CarerQoL-VAS ‘transfer’) was added, and informal caregivers were asked to estimate their general well-being in the hypothetical situation that all informal care activities were to be passed on to another, self-selected person. We calculated the difference between these VAS scores to explore whether informal caregivers derived happiness from providing informal care (so-called process utility). The construct validity of the CarerQoL-7D instrument was validated in different study settings (i.e., the general population, hospitals, long-term care facilities and primary care centers) [21–23].

#### EQ-5D

The Euroqol-5 Dimensions using 3 levels (EQ-5D) was used in the BIOS to measure HS of the hip-fracture patient [24]. This generic health utility instrument consists of five dimensions (mobility, self-care, usual activities, pain/discomfort, and anxiety/depression) with 3 levels each (none, some or many limitations). The Dutch tariff was used to obtain utilities [25, 26]. The EQ-5D is a valid and reliable instrument and can be used as an outcome measure for patients recovering from a hip fracture [25–27].

#### GFI

The Groningen Frailty Index (GFI) was used in the BIOS to evaluate the level of frailty of the patient [28–30]. The GFI is a 15-item self-reported instrument and measures the loss of functions and capabilities in four domains: physical, cognitive, social and mental functioning. The sum score of the GFI ranges from 0 to 15, with a score of ≥4 indicating frailty. The GFI is a valid, reliable and feasible instrument for use with elderly people living either at home or in an institution to detect those who are at a high risk of a poor outcome [29, 30].

### Data analysis

We calculated descriptive statistics to assess caregivers’ and patients’ characteristics. We expressed continuous variables as a mean with standard deviation and categorical variables as numbers and percentages. We described the nature and intensity of informal care provided by caregivers, as expressed by hours of care per week and types of activities. We evaluated the CarerQoL-7D score, CarerQoL-VAS, CarerQoL-VAS ‘transfer’ and process utility at one, three and six months. We used univariate linear regression analysis to assess whether caregivers’ or patients’ characteristics influenced the intensity of informal care or the CarerQoL of the informal caregiver. We built a multivariable linear regression model to determine the association between independent caregivers’ and patients’ characteristics and dependent variables, intensity of provided informal care and CarerQoL of the informal caregiver, adjusted for different covariates. Different covariates were clinically relevant variables from both caregivers’ and patients’ characteristics, such as hours of informal care, partner, caregiver age, caregiver sex, caregiver educational attainment, patient age, living in an institution, dementia and measurement time points. Finally, we built a multivariable logistic regression model to examine how caregivers’ and patients’ characteristics are associated with the dimensions of the CarerQol-7D, adjusted for the covariates partner, caregiver age, caregiver sex, living in an institution, dementia and GFI. Regression coefficients (*β*), adjusted odds ratios (aOR) and 95% confidence intervals (95% CI) were calculated. All analyses were conducted with SPSS version 24 (IBM SPSS Statistics for Windows, Armonk, NY, USA), and a *p*-value < 0.05 was considered statistically significant.

## Results

### Response

In total, 255 contact persons for patients with a hip fracture were approached by telephone. Forty-nine persons were excluded; of thee, 29 contact persons stated that they had never had to provide informal care, in 11 cases no informal caregiver was available, and nine patients had passed away by the time of the call. In total, 206 caregivers were eligible for inclusion. A total of 78 persons could not be reached, despite repeated telephone calls, and five caregivers expressed no interest. No significant difference was found in patient demographics (age: *p* = 0.29; sex: *p* = 0.63) between responders and non-responders. Table 1 provides caregiver and patient characteristics for the study population. In total, 123 informal caregivers who provided informal care to 123 hip fracture patients were included. (response: 59.7%). Forty, 39 and 44 informal caregivers were included, respectively, in the groups approached at one, three or six months after a hip fracture was suffered by their loved one. The mean age of the caregivers was 64.6 years and 55.3% were female. The patients’ mean age was 79.9 years, and 74.0% were female. Patients had a mean total GFI score of 10.7 and were all considered to be frail. In the group of caregivers providing informal care at one month after hip fracture, there were no patients with dementia or patients who, pre-fracture, were living in an institution.

**Table 1:** Characteristics of informal caregivers and patients after a hip fracture

*Caregiver characteristic*	Total (*n* = 123)	1 month (*n* = 40)	3 months (*n* = 39)	6 months (*n* = 44)
Age in years (M,SD)	64.6 (12.2)	67.6 (11.0)	64.7 (12.2)	61.9 (12.9)
Female sex (N,%)	68 (55.3)	22 (55)	22 (56.4)	24 (54.5)
Relationship (N,%)				
*Partner*	55 (44.7)	27 (67.5)	15 (38.5)	13 (29.5)
*Child*	53 (43.1)	9 (22.5)	20 (51.3)	24 (54.5)
*Sibling*	7 (5.7)	2 (5.0)	2 (5.1)	3 (6.8)
*Other*	8 (6.5)	2 (5.0)	2 (5.1)	4 (9.1)
Educational attainment^a^ (N,%)				
*Low*	37 (30.1)	11 (27.5)	15 (38.5)	11 (25)
*Middle*	56 (45.5)	21 (52.5)	15 (38.5)	20 (45.5)
*High*	30 (24.4)	8 (20.0)	9 (23.0)	13 (29.5)
*Patient characteristic*				
Age in years (M,SD)	79.9 (8.3)	77.6 (8.1)	79.3 (8.7)	82.6 (7.3)
Female sex (N,%)	91 (74.0)	27 (67.5)	29 (74.4)	35 (79.5)
Dementia; yes (%)	22 (17.9)	0 (0.0)	8 (20.5)	14 (31.8)
Pre-fracture living in an institution	17 (13.8)	0 (0.0)	5 (12.8)	12 (27.3)
Discharge to home^b^; yes (%)	59 (55.7)	22 (75.9)	18 (48.6)	19 (47.5)
Pre-fracture mobility^b^ (N,%)				
*Freely mobile without aids*	57 (54.8)	27 (75.0)	18 (51.4)	12 (36.4)
*Mobile with aids*	44 (42.3)	9 (25.0)	16 (45.7)	19 (57.6)
*No functional mobility*	3 (2.9)	0 (0.0)	1 (2.9)	2 (6.0)
Type of treatment (N,%)				
*Nonoperative*	2 (1.6)	1 (2.5)	1 (2.6)	0 (0.0)
*Intramedullary fixation*	47 (38.2)	11 (27.5)	19 (48.7)	17 (38.6)
*Cannulated screws*	12 (9.8)	6 (15.0)	5 (12.8)	1 (2.3)
*Hemi-arthroplasty*	49 (39.8)	17 (42.5)	10 (25.6)	22 (50.0)
*Total hip arthroplasty*	13 (10.6)	5 (12.5)	4 (10.3)	4 (9.1)
Length of hospital stay (M,SD)	8.6 (5.0)	7.3 (3.6)	9.5 (5.7)	9.0 (5.3)
Comorbidity				
*None*	19 (15.4)	10 (25.0)	4 (10.3)	5 (11.4)
*One*	45 (36.6)	12 (30.0)	17 (43.6)	16 (36.4)
*Two or more*	59 (48.0)	18 (45.0)	18 (46.2)	23 (52.3)
Post-fracture mobility (N,%)				
*Freely mobile without aids*	17 (13.8)	1 (2.5)	6 (15.4)	10 (22.7)
*Mobile with aids*	84 (68.3)	27 (67.5)	28 (71.8)	29 (65.5)
*No functional mobility*	22 (17.9)	12 (30.0)	5 (12.8)	5 (11.4)
EQ-5D (M,SD)	0.53 (0.27)	0.57 (0.26)	0.52 (0.28)	0.50 (0.28)
GFI (M,SD)	10.7 (2.9)	9.8 (1.8)	9.9 (2.3)	12.3 (3.3)

*Abbreviations*: *M* mean, *SD* standard deviation, *n* number of caregivers, *EQ-5D* Euroqol-5 Dimensions, *GFI* Groningen Frailty Indicator

^a^Educational attainment: Low = no diploma, primary education, preparatory secondary vocational education; Middle = university preparatory education, senior general secondary education, senior secondary vocational education and training; High = universities of applied sciences: associate degree or university degree

^b^Number of missing values: discharge to home: *n* = 17; pre-fracture mobility: *n* = 19

### Intensity

On average, informal caregivers provided 39.5 h (SD 32.8) of informal care per week for the first six months after a hip fracture, which differed significantly between the measurement time points (*p* ≤ 0.01). At one, three and six months after the hip fracture, this figure was 50.3 (SD 32.1), 45 (SD 38.2) and 25 (SD 21.7) hours per week, respectively (Table 2). Around half of the informal care activities consisted of providing additional social support, and approximately 20% of the activities involved carrying out household chores.

**Table 2:** Intensity of informal care provided by nature of care for hip fracture patients and CarerQoL-score

	Total (*n* = 123)	1 month (*n* = 40)	3 months (*n* = 39)	6 months (*n* = 44)	p
Total hours per week of informal care (M,SD)	39.5 (32.8)	50.3 (32.1)	45.0 (38.2)	24.8 (21.7)	< 0.01
Nature of informal care activities *(% of total hours)*					
- Personal care	8.6	9.1	9.4	6.3	
- Household chores	19.7	20.1	18.7	20.5	
- Nursing	1.4	3.1	0.2	0	
- Mobility outdoors	9.1	7.7	7.9	13.5	
- Logistic activities	4.5	2.8	4.4	7.7	
- Social activities	56.8	57.2	59.4	52.0	
CarerQoL-7D score (M,SD)	83.7 (15.0)	81.6 (16.7)	87.0 (12.8)	82.6 (15.0)	0.23
CarerQoL-VAS (M,SD)	7.6 (1.5)	7.3 (1.8)	7.9 (1.1)	7.5 (1.3)	0.13
CarerQoL-VAS ‘transfer’ (M,SD)	6.8 (2.1)	6.5 (2.0)	6.7 (2.4)	7.2 (1.9)	0.26
Process utility (M,SD)	0.7 (2.0)	0.8 (2.0)	1.2 (2.3)	0.27 (1.71	0.11

*Abbreviations*: *M* mean, *SD* standard deviation, *n* number of caregivers

Table 3 shows the univariate and multivariable linear regression analysis. Univariate analysis shows that caregiver characteristics such as being a partner (*β* 42.5), age (*β* 1.3) and educational attainment (middle vs. low *β* -17.0 and high vs. low *β* -27.4) were significantly associated with the intensity of informal care provided. Patient characteristics such as age (*β* -1.4), living in an institution (*β* -20.6), dementia (*β* -21.5) and GFI (*β* -2.9) were also significantly associated with intensity of informal care provided. In the multivariable analyses, the intensity of care provided was not significantly explained by patient or caregiver characteristics, except for the relationship with the patient: if the informal caregiver was the patient’s partner, the intensity of informal care was 34.0 h per week higher over the first six months after hip fracture compared to a non-partner (95% CI 20.9-47.1).

**Table 3:** Univariate- and multivariable linear regression results for association with intensity of provided informal care of informal caregivers

	Unadjusted^a^			Adjusted^b^		
*Caregiver characteristic*	*β*	95% CI	p	*β*	95% CI	p
CarerQoL	−0.1	−0.5 – 0.3	0.60	−0.2	− 0.5 – 0.1	0.19
Partner	42.5	33.4 – 51.5	< 0.001	34.0	20.9 – 47.1	< 0.001
Age	1.3	0.9 – 1.7	< 0.001	0.3	−0.2 – 0.8	0.23
Female sex	−11.8	−23.5 – −0.2	0.05	−4.6	−13.8 – 4.7	0.33
Educational attainment						
* Middle* vs. *low*	−17.0	−30.2 – − 3.8	0.01	−2.4	− 13.5 – 8.6	0.66
* High* vs. *low*	−27.4	−42.7 – −12.1	0.001	−9.1	−21.9 – 3.6	0.16
Measurement time point						
* At 3 months* vs. *1 month*	−5.4	−19.2 – 8.5	0.45	6.7	−4.9 – 18.4	0.26
* At 6 months* vs. *1 month*	−25.6	−39.0 – 12.1	< 0.001	−8.3	− 20.1 – 3.5	0.16
*Patient characteristic*						
Age	−1.4	−2.1 – −0.7	< 0.001	0.02	-0.7 – 0.7	0.95
Female sex	−2.0	−15.4 – 11.4	0.77	8.9	−1.7 – 19.5	0.10
Mobility						
* Some problems* vs. *no problems*	12.0	−5.2 – 29.2	0.17	2.7	−10.8 – 16.1	0.39
* Confined to bed* vs. *no problems*	19.0	−1.8 – 39.9	0.07	5.1	−11.7 – 21.9	0.60
Living in an institution	−20.6	−37.2 – −3.9	0.02	−0.3	− 20.8 – 21.5	0.97
Dementia	−21.5	−36.4 – −6.6	< 0.01	−7.0	− 26.5 – 12.6	0.48
GFI	−2.9	−5.1 – −0.6	0.01	1.9	−0.4 – 4.1	0.10
EQ-5D	10.9	−16.6 – 38.4	0.43	−10.4	−32.2 – 11.4	0.35

*Abbreviations*: *CI* confidence interval, *CarerQoL* care-related quality of life, *EQ-5D* Euroqol-5 Dimensions, *GFI* Groningen Frailty Indicator

^a^Univariate linear regression analysis

^b^Multivariable linear regression analysis, adjusted for: partner, caregiver age, caregiver gender, caregiver educational attainment, patient age, living in an institution, dementia and measurement time points

### CarerQoL and process utility

**Fig. 1:**
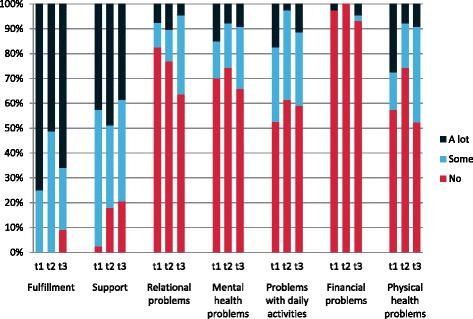
Distribution of CarerQoL-7D – dimensions reported by informal caregivers providing informal care at 1, 3 and 6 months (t1,t2,t3) after hip fracture

The CarerQoL-7D score was averaged over three measurement time points, 83.7 (SD 15), and did not show any significant differences between the different time points (Table 2). Informal caregivers estimated their general well-being at 7.6 (1.5) on average on the CarerQoL-VAS (Table 2). The CarerQoL-VAS ‘transfer’ was significantly lower (*p* < 0.001) with an average of 6.8 (2.1), which meant that the process utility measured for the 123 informal caregivers was positive; informal caregivers derive happiness from providing care and would be unhappier if care was transferred to another person. In total, 31.1% had a positive, 48.4% a neutral and 20.5% a negative process utility. No significant differences between the measurement time points were noted for process utility (*p* = 0.11). Figure 1 shows the distribution of responses across the seven domains of the CarerQoL-7D. Almost all the informal caregivers stated that they gained some or a lot of satisfaction from providing informal care (irrespective of time point). The majority did not experience financial problems due to caregiving. At one, three and six months, 42.5%, 25.6% and 47.5%, respectively, experienced some to a lot of physical health problems. Some to a lot of mental problems occurred in 30%, 25.6% and 34.1% of caregivers, respectively. In addition, 47.5%, 38.5% and 40.9% reported some to a lot of problems with combining informal care and their own daily activities for the three time points. Informal caregivers who provided more hours of informal care complained significantly more often about physical health problems (*p* = 0.01). Most of the informal caregivers received some or a lot of support from others in providing informal care.

Table 4 shows that female informal caregivers (55.3%) had a significantly lower CarerQoL-7D score in both uni- and multivariable regression analysis (adjusted *β* -7.8; 95% CI: -13.3 – -2.3). Multivariable linear regression showed no other significant characteristics associated with the CarerQoL-7D score. Caregiver characteristics including age and female sex were associated with relational problems (Table 5). In multivariable models, female caregivers were 3.0 times more likely to experience relational problems (aOR 3.02; 95% CI 1.08-8.43). Caregivers providing care at 6 months were associated with physical health problems (aOR 2.54; 95% CI 1.05-6.14). Dementia was also associated with relational problems (aOR 8.25; 95% CI, 1.35-50.48).

**Table 4:** Univariate- and multivariable linear regression results for association with CarerQoL of informal caregivers

	Unadjusted^a^			Adjusted^b^		
*Caregiver characteristic*	*β*	95% CI	p	*β*	95% CI	p
Hours of informal care	−0.02	− 0.1 – 0.06	0.60	− 0.05	−0.1 – 0.06	0.38
Partner	0.5	−4.9 – 5.9	0.85	5.3	−3.3 – 13.9	0.23
Age	0.0	−0.2 – 0.2	0.97	0.02	−0.2 – 0.3	0.91
Female sex	−7.1	−12.4 – − 1.9	0.01	− 7.8	− 13.3 – − 2.3	0.01
Educational attainment						
* Middle* vs. *low*	4.9	−1.3 – 11.2	0.12	5.8	−0.7 – 12.3	0.08
* High* vs. *low*	5.3	−1.9 – 12.6	0.15	5.5	−2.1 – 13.1	0.16
Measurement time point						
* At 3 months* vs. *1 month*	5.4	−1.2 – 12.1	0.11	6.3	−0.3 – 12.9	0.06
* At 6 months* vs. *1 month*	1.0	− 5.4 – 7.4	0.76	0.6	−6.2 – 7.4	0.86
*Patient characteristic*						
Age	−0.04	−0.4 – 0.3	0.82	−0.2	−0.5 – 0.2	0.36
Female sex	3.2	−2.9 – 9.3	0.30	1.4	−4.8 – 7.7	0.65
Mobility						
* Some problems* vs. *no problems*	4.0	−3.9 – 11.9	0.32	5.4	−2.4 – 13.3	0.17
* Confined to bed* vs. *no problems*	5.8	−3.7 – 15.4	0.23	8.3	−1.5 – 18.1	0.10
Living in an institution	3.3	−4.4 – 11.0	0.40	3.8	−4.3 – 11.9	0.36
Dementia	0.4	−6.6 – 7.4	0.91	−0.04	−7.6 – 7.5	0.99
GFI	−0.9	−2.0 – 0.13	0.09	−0.4	−1.6 – 0.9	0.57
EQ-5D	3.0	−8.4 – 14.5	0.60	2.9	−9.1 – 14.9	0.63

*Abbreviations*: *CarerQoL* care-related quality of life, *CI* confidence interval, *EQ-5D* Euroqol-5 Dimensions, *GFI* Groningen Frailty Indicator

^a^Univariate linear regression analysis

^b^Multivariable linear regression analysis, adjusted for: hours of informal care, caregiver age, caregiver gender, caregiver educational attainment, patient age and measurement time points

**Table 5:** Multivariable logistic regression results for association of dimensions of the CarerQol of informal caregivers

	Adjusted odds ratio (95% CI)^a^					
*Caregiver characteristic*	Fulfillment^b^	Support^b^	Relational problems^c^	Mental health problems^c^	Problems with daily activities^c^	Physical health problems^c^
Hours of informal care	1.00 (0.98 – 1.01)	1.00 (0.98 – 1.01)	1.00 (0.98 – 1.03)	1.01 (0.99 – 1.03)	1.02 (1.00 – 1.03)	1.01 (1.00 – 1.03)
Hours of informal care						
* > 25 h*	1.61 (0.59 – 4.43)	0.92 (0.35 – 2.41)	2.13 (0-.61 – 7.43)	3.43 (1.10 – 10. 64)	1.24 (0.47 – 3.30)	1.62 (0.61 – 4.29)
* > 40 h*	1.10 (0.37 – 3.30)	1.07 (0.38 – 3.03)	3.00 (0.68 – 13.35)	3.17 (0.82 – 12.27)	2.26 (0.74 – 6.92)	1.77 (0.62 – 5.05)
Partner	1.06 (0.39 – 2.87)	1.82 (0.74 – 4.48)	0.41 (0.13 – 1.28)	0.42 (0.15 – 1.17)	1.24 (0.50 – 3.06)	2.30 (0.92 – 5.73)
Age	0.98 (0.94 – 1.03)	0.99 (0.96 – 1.03)	1.07 (1.01 – 1.12)	1.02 (0.97 – 1.06)	1.00 (0.96 – 1.04)	1.00 (0.96 – 1.04)
Female sex	0.43 (0.18 – 0.98)	0.87 (0.40 – 1.88)	3.02 (1.08 – 8.43)	1.61 (0.67 – 3.85)	1.86 (0.84 – 4.12)	0.96 (0.43 – 2.12)
Educational attainment						
* Low*	1 [Reference]	1 [Reference]	1 [Reference]	1 [Reference]	1 [Reference]	1 [Reference]
* Middle*	1.29 (0.51 – 3.27)	0.78 (0.32 – 1.91)	0.81 (0.26 – 2.51)	0.80 (0.29 – 2.17)	0.78 (0.31 – 1.99)	0.49 (0.20 – 1.23)
* High*	2.27 (0.72 – 7.21)	0.72 (0.25 – 2.07)	1.12 (0.30 – 4.11)	0.50 (0.14 – 1.72)	2.06 (0.71 – 6.01)	0.45 (0.15 – 1.35)
Measurement time point						
* 1 month*	1 [Reference]	1 [Reference]	1 [Reference]	1 [Reference]	1 [Reference]	1 [Reference]
* 3 months*	0.62 (0.37 – 1.04)	1.36 (0.53 – 3.50)	0.75 (0.20 – 2.78)	0.73 (0.25 – 2.17)	0.83 (0.36 – 1.88)	0.48 (0.20 – 1.14)
* 6 months*	0.80 (−.33 – 1.90)	0.85 (0.32 – 2.30)	1.58 (0.46 – 5.46)	0.92 (0.31 – 2.73)	0.78 (0.33 – 1.83)	2.54 (1.05 – 6.14)
*Patient characteristic*						
Age	1.00 (0.94 – 1.06)	0.97 (0.92 – 1.03)	0.99 (0.92 – 1.06)	0.97 (0.91 – 1.04)	0.99 (0.94 – 1.05)	1.06 (1.01 – 1.13)
Female sex	1.18 (0.46 – 3.00)	1.02 (0.42 – 2.48)	0.29 (0.10 – 0.90)	0.80 (0.30 – 2.19)	0.54 (0.21 – 1.34)	1.43 (0.55 – 3.75)
Mobility						
* No problems*	1 [Reference]	1 [Reference]	1 [Reference]	1 [Reference]	1 [Reference]	1 [Reference]
* Some problems*	0.80 (0.06 – 10.87)	0.88 (0.29 – 2.67)	0.56 (0.16 – 1.95)	0.44 (0.14 – 1.37)	1.80 (0.55 – 5.78)	2.39 (0.68 – 8.41)
* Confined to bed*	2.71 (0.40 – 18.57)	1.24 (0.33 – 4.65)	0.31 (0.06 – 1.72)	0.22 (0.05 – 1.01)	1.23 (0.30 – 5.04)	1.63 (0.37 – 6.89)
Living in an institution	7.00 (0.76 – 64.61)	1.72 (0.28 – 10.51)	0.56 (0.07 – 4.47)	0.31 (0.04 – 2.34)	0.21 (0.03 – 1.58)	0.78 (0.10 – 6.06)
Dementia	0.63 (0.12 – 3.38)	1.27 (0.26 – 6.21)	8.25 (1.35 – 50.48)	0.74 (0.13 – 4.13)	2.71 (0.47 – 15.69)	0.45 (0.08 – 2.73)
GFI	1.01 (0.85 – 1.19)	0.96 (0.82 – 1.12)	1.08 (0.90 – 1.29)	1.22 (1.03 – 1.46)	1.14 (0.98 – 1.34)	1.14 (0.97 – 1.35)

*Abbreviations*: *CarerQoL* care-related quality of life, *CI* confidence interval, *GFI* Groningen Frailty Indicator

*Note*: due to small numbers of caregivers experienced financial problems, no associations within this domain were given in this table

^a^Adjusted for: partner, caregiver age, caregiver gender, living in an institution, dementia and GFI

^b^Fulfilment and support related to providing care, with contrast between caregivers who rated the level of fulfilment and support of no or some vs caregivers who indicated a lot fulfilment and support

^c^Relational problems, mental health problems, problems with daily activities, physical health problems, with contrast between caregivers who rated the level of no problems vs caregivers who indicated some or a lot problems

## Discussion

To our knowledge, this is the first study to examine CarerQoL in informal caregivers of patients aged 65 years and older after a hip fracture. We used the CarerQoL-7D, which characterizes burden across seven dimensions of burden with individual weighted scores.

Our findings contribute important insight regarding the ‘invisible work’ of managing care during the first six months after the hip fracture of a loved one, confirmed by the great intensity of provided informal care with a mean of 39.5 h per week. This study identifies higher-intensity caregivers, who are largely unrecognized in our healthcare system. Partners provided significantly more hours of informal care per week compared to other types of caregivers, but they showed no difference in CarerQoL-scores (*β* 5.3; 95% CI -3.3-13.9).

The median CarerQoL-7D score (83.7) found in this study is similar to that in earlier CarerQoL-7D studies [31, 32]. Hoefman et al. and Van Dam and colleagues examined the CarerQoL (79.1 and 83.9, respectively) of informal caregivers in a heterogeneous patient population that was representative of the Dutch population. Our finding confirms the assumption that there is no significant association between age and CarerQoL of the informal caregiver. In contrast to our study, they revealed a significant association between patients with impaired cognition and a lower CarerQoL-score. However, when focused on the domain ‘relational problems’, we found a significant association between dementia and some or many relational problems experienced by the caregiver. Wolf et al. found in a representative study that almost half of their investigated caregivers provided substantial help with health care activities when assisting an older adult with dementia [33]. They found that caregivers who provided care to patients with both dementia and severe disability were 1.8 times more likely to experience emotional difficulty (95% CI 1.10-2.87).

Caregiver literature has consistently shown that female caregivers are more burdened than male caregivers [34–36]. Males and females experience caregiving differently, and explanations of sex differences in caregiver burden are that males and females live in different structural contexts, which leads to different kinds and intensities of stressors to which people are exposed. In addition, females mostly provide more hours of informal care, experience more negative effects of caregiving and are more sensitive to a feeling of distance between themselves and the person being cared for [35, 36]. This might result in a loss of self-esteem, which can ultimately lead to depression [37]. However, in contrast with this theory, we found no difference in the domain of mental problems between male and female informal caregivers. Additionally, and in contrast to Van Dam et al., we found that female informal caregivers stated a significantly lower level of CarerQoL (*β* -7.8; 95% CI: -13.3 – -2.3) and were 3.00 times more likely to experience relational problems. Surprisingly, we found no significant difference in the intensity of informal care provided by men and women. A possible explanation could be the type of this elderly, predominantly female hip-fracture population for whom caregivers provided informal care in this study. In total 44.7% of the caregivers were male and had to provide a great deal of intense informal care to a loved one with a hip fracture. In addition, almost 25% were male partners with a mean age of 68.8 years (versus 61.2 years for women). This may have led to more equality in the intensity of provided informal care between men and women.

Based on the unadjusted analyses, the intensity of provided informal care was significantly lower for older patients, patients with dementia, patients with a higher GFI and patients already residing in an institution before the fracture. In the Dutch healthcare system, more frail patients and more patients with dementia reside in nursing homes. They receive more formal care, which could be one of the possible explanations why these patients with a hip fracture require fewer hours of informal care than do elderly patients with a hip fracture residing in the community.

Around half of the patients were discharged to their homes after treatment in the hospital, and subsequent informal care was often imposed on the partner. A common remark during telephone interviews was that they received little or no information in advance about this sudden new ‘task’ of great intensity, according to our results about the intensity of care provided by partners (*β* 34.0; 95% CI 20.9 - 47.1).

This study showed that up to 26.0% of the informal caregivers experienced some or many relational problems, 34.1% experienced some or a lot of mental health problems, and 47.5% experienced physical health problems. These problems experienced by those providing informal care can be eased with careful attention from healthcare professionals. Schulz et al. stated that counseling, relaxation training, and respite programs can improve caregiver quality of life by increasing caregiver abilities and confidence to manage daily care challenges [38]. These interventions may delay and reduce the care recipient’s institutionalization and reduce re-hospitalization [39, 40]. Therefore, we recommend that it is better to inform prospective informal caregivers of patients with a hip fracture about their task at an early stage in the hospital setting. Another important aspect that applies particularly in the case of a patient with a hip fracture is properly educating informal caregivers about the expected course of recovery [17]. Naturally, this varies from patient to patient. The goal of the recovery after a hip fracture is to restore the previous level of ADL. In practice, however, there is a considerable gap between this goal and reality. First, the high mortality of up to 30% in the general population in the first year must not be underestimated [13]. Second, the level of frailty in the aging population is increasing, and there is a delicate balance between the physical, cognitive and social aspects [41]. Our study showed that all participants receiving a total GFI score of 4 and above and were frail. Problems tend to persist in this growing group of elderly with a hip fracture in terms of poorer conditioning with decreased mobility and reduced QoL [14]. Providing realistic expectations for recovery when educating patients and their informal caregivers can help. Nahm et al. reported that informal caregivers often state that their loved one does not get the right kind and amount of care and rehabilitation in the rehabilitation environment [17]. Given this mismatch, informal caregivers must be better informed about the goal of rehabilitation, which is to assist patients with their recovery, and about the role of informal caregivers, which is to motivate their loved one to do the exercises themselves or assisted by others.

As in any survey, the results are subject to the constraints of sample design, participant response, variables asked, and outcomes used. Because this a cross-sectional survey in which informal caregivers were not followed over time, we are unable to comment on the causal processes that underlie the observed CarerQoL. When interpreting the results of the three groups, heterogeneity of the groups must be considered. The number of contact persons approached by telephone who stated that they no longer needed to provide informal care increased in the three- and six-month groups. This finding suggests that the group still receiving informal care at six months is an older and frailer group, in which the number of patients with dementia, the number of patients who had been living in an institution before their hip fracture and the GFI score are higher (Table 1). Another limitation is that non-response bias cannot be excluded in this study because no demographic data could be collected for contact persons (potential caregivers) who could not be reached by telephone. We could have missed informal caregivers who were too busy or perhaps overburdened so that they were not at home at the time of our call; therefore, our results must be interpreted with caution. However, we randomly selected a subset of individuals, and we discovered no significant difference in patient demographics between responders and non-responders. Response bias could also have had an impact on how caregivers completed the CarerQoL-VAS and the Carer-QoL-VAS ‘transfer’ because we administered our results by phone and we verbally asked for a score between 0 (completely unhappy) and 10 (completely happy). It could be possible that caregivers provide a socially desirable response that may affect the response in some way [42].

A strength of this study is the use of the CarerQoL-7D instrument to measure the CarerQoL of the informal caregivers. In contrast to the first limitation given above, a cross-sectional study is the primary source of evidence for measuring this construct. The great benefit of the CarerQoL-7D instrument over earlier studies that measured the burden on informal caregivers is the fact that it can measure positive dimensions as well as the burden, such as satisfaction and support received from others. In this study, informal caregivers experienced considerable support and satisfaction, in agreement with informal caregivers in other populations [31]. In total, 79.5% of caregivers stated that their well-being would remain the same or even decrease if they could give the informal care tasks free of charge to another person chosen by them and the patient, despite the time investment and mental and physical burden of informal care. This is also reflected in the result from the ‘satisfaction’ and ‘support’ domains in the CarerQoL-7D. Another strength, in contrast with Van Dam et al., is that we included caregivers from a homogenous group of 123 hip fracture patients. As we mentioned before, an important aspect is to properly educate informal caregivers about the expected course of recovery. This aspect depends on the study population and is more difficult in a heterogeneous geriatric population that includes stroke, elective, trauma and other patients than in our study, which included caregivers of patients with a hip fracture.

To examine in more detail the course of the burden on informal caregivers for patients with a hip fracture, expressed by the intensity of provided informal care and the CarerQoL, it would be valuable to conduct a prospective observational study. An advantage of this study would be that one can follow change over time in particular individuals within a cohort. This would enable us to relate CarerQoL to particular exposures and to further define these exposures with regards to presence, timing and chronicity. This could help healthcare providers to focus more on caregiver CarerQoL, with attention to physical- and mental health problems that informal caregivers frequently report.

## Conclusion

Informal caregivers, especially partners, are faced with providing care of greater intensity to elderly patients during the first six months after a hip fracture. The CarerQoL was not associated with the intensity of the provided informal care. As the Dutch healthcare system undergoes reform, the pressure on informal caregivers will only increase. This study shows that a considerable group of informal caregivers for elderly patients with a hip fracture experienced relational, physical and mental health problems that stemmed from providing intensive informal care during the first six months.

## Additional file

Additional file 1: Care-related Quality of Life instrument. (DOCX 131 kb)

## Abbreviations

ADL: Activities of daily living
aOR: Adjusted odds ratio
BIOS: Brabant Injury Outcome Surveillance
CarerQoL: Care-related Quality of Life
CI: Confidence interval
EQ-5D: EuroQol-5 Dimensions
GFI: Groningen Frailty Indicator
HS: Health status
QoL: Quality of Life
SD: Standard Deviation
VAS: Visual analogue scale
