# Supplementary figures and images for: Care-related Quality of Life of informal caregivers of the elderly after a hip fracture
Source: J Patient Rep Outcomes. 2018 May 3;2:23. doi: 10.1186/s41687-018-0048-3 (PMC5934924; doi:10.1186/s41687-018-0048-3)

**Additional file 1:** Care-related Quality of Life instrument


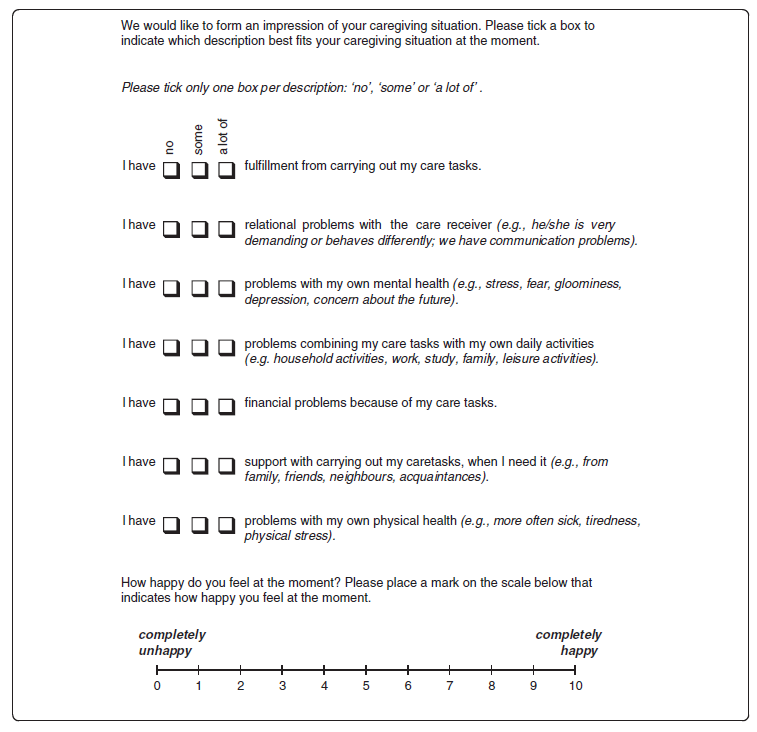

Supplement: Supplementary file 1 — Care-related Quality of Life instrument. (DOCX 131 kb) [file 41687_2018_48_MOESM1_ESM.docx]
